# Whole-grain food consumption in Singaporean children aged 6–12 years

**DOI:** 10.1017/jns.2016.25

**Published:** 2016-08-04

**Authors:** Jia En Neo, Saihah Binte Mohamed Salleh, Yun Xuan Toh, Kesslyn Yan Ling How, Mervin Tee, Kay Mann, Sinead Hopkins, Frank Thielecke, Chris J. Seal, Iain A. Brownlee

**Affiliations:** 1 Human Nutrition Research Centre, School of Agriculture, Food & Rural Development, Newcastle University, Singapore; 2 Institute of Health and Society, Newcastle University, Newcastle upon Tyne, UK; 3 Cereal Partners Worldwide, Lausanne, Switzerland; 4 Nestlé Research Centre, Vers-chez-les-Blanc, Lausanne, Switzerland; 5 Human Nutrition Research Centre, School of Agriculture, Food & Rural Development, Newcastle University, Newcastle upon Tyne, UK

**Keywords:** Whole grains, Singapore, Children, Dietary intake, HPB, Health Promotion Board, IQR, interquartile range, RTEBC, ready-to-eat breakfast cereal

## Abstract

Public health bodies in many countries are attempting to increase population-wide habitual consumption of whole grains. Limited data on dietary habits exist in Singaporean children. The present study therefore aimed to assess whole grain consumption patterns in Singaporean children and compare these with dietary intake, physical activity and health parameters. Dietary intake (assessed by duplicate, multipass, 24-h food recalls), physical activity (by questionnaire) and anthropometric measurements were collected from a cross-section of 561 Singaporean children aged 6–12 years. Intake of whole grains was evaluated using estimates of portion size and international food composition data. Only 38·3 % of participants reported consuming whole grains during the dietary data collection days. Median intake of whole grains in consumers was 15·3 (interquartile range 5·4–34·8) g/d. The most commonly consumed whole-grain food groups were rice (29·5 %), wholemeal bread (28·9 %) and ready-to-eat breakfast cereals (18·8 %). A significantly lower proportion of Malay children (seven out of fifty-eight; *P* < 0·0001) consumed whole grains than children of other ethnicities. Only 6 % of all children consumed the amount of whole grains most commonly associated with improved health outcomes (48 g/d). There was no relationship between whole grain consumption patterns and BMI, waist circumference or physical activity but higher whole grain intake was associated with increased fruit, vegetable and dairy product consumption (*P* < 0·001). These findings demonstrate that consumption of whole grain foods is low at a population level and infrequent in Singaporean children. Future drives to increase whole-grain food consumption in this population are likely to require input from multiple stakeholders.

Previous observational evidence has consistently suggested that increasing whole-grain food intake is associated with reduced risk of CVD, type 2 diabetes, obesity^(^^1^^,^^2^^)^ and some types of cancer^(^^3^^,^^4^^)^ as well as a reduction in overall mortality^(^^5^^–^^7^^)^. These findings remain the cornerstone for public health activities initiated by academic groups to increase intake of whole grains. Recently, a series of observational and intervention studies carried out in Asian populations corroborated the reduction in disease risk with the consumption of whole grains^(^^8^^)^. For example, in a randomised controlled trial of forty-four overweight or obese female children from Iran, consumption of whole-grain foods (at least half of their total servings of grains) for 6 weeks was shown to lower markers of systemic inflammation. Consumption of oatmeal *v*. wheat noodles (100 g/d of either) for 6 weeks decreased major risk factors for CVD in Chinese adults^(^^9^^)^ and in Singaporean Chinese adults replacing one serving of rice with whole wheat bread was associated with a lower risk of IHD mortality^(^^10^^)^. More generally, whole grains as part of a healthy dietary pattern have been shown to moderate the risk of diabetes and obesity in Mongolian and Korean adults^(^^11^^,^^12^^)^. These studies highlight that increasing whole grain intake is likely to benefit public health in Asian populations.

Dietary guidelines promoting the increased consumption of whole grains exist in some countries worldwide^(^^13^^)^. Longitudinal dietary intake data from the USA suggest a small trend for increasing intake in this population^(^^14^^)^; however, current intakes are still well below the recommended levels (three portions or 48 g/d)^(^^15^^)^. There are limited data on trends in wholegrain intake worldwide. Singapore is a rapidly developed island nation with a unique food culture^(^^16^^)^. The local public health agency (The Health Promotion Board; HPB) has recently developed recommendations for inclusion of more whole-grain foods (based on suggested servings equivalent to half a bowl of brown rice or two slices of wholemeal bread, two chapattis, two-thirds of a bowl of uncooked oats or four whole-wheat biscuits) within daily dietary intake^(^^17^^,^^18^^)^. The HPB has a number of current campaigns targeting increasing whole-grain food intake at a population level, particularly through out-of-home food consumption occasions^(^^19^^)^. Similar to other parts of the world, Singaporean National Nutrition Survey data suggest that, although dietary intake of whole grains is less than recommended, the percentage of adults consuming one or more servings of whole grains has increased^(^^20^^)^ from 8·4 % in 2004 to 27·0 % in 2010.

The most recent Singaporean National Nutrition Survey only collected information on dietary intake from adults (aged 18–69 years). The data suggest that those in lower adult age categories tend to consume whole grains less frequently than older Singaporeans. Findings from other countries suggest that dietary intake of whole grains is lower in children and adolescents than other sections of the population^(^^21^^–^^24^^)^. While development of habitual food choice is complex, previous research suggests that many dietary habits are developed at a young age and may persist into adulthood^(^^25^^–^^28^^)^. Therefore, attempting to improve dietary habit through increased whole-grain food consumption at an early age has the potential to lead to improvements in lifelong health and wellness. Within the Singaporean context, it is important to understand current dietary intake, not only to consider the current situation in young Singaporeans, but also to help develop future strategies for whole-grain food producers and public health agencies to target increased and lifelong intake of whole grains.

The aim of the present study was to assess whole-grain food consumption patterns in Singaporean children aged 6–12 years old. In addition, our objectives were to investigate whether whole grain food consumption was linked to overall dietary habits and with simple estimates of adiposity.

## Materials and methods

### Study population

**Fig. 1.**
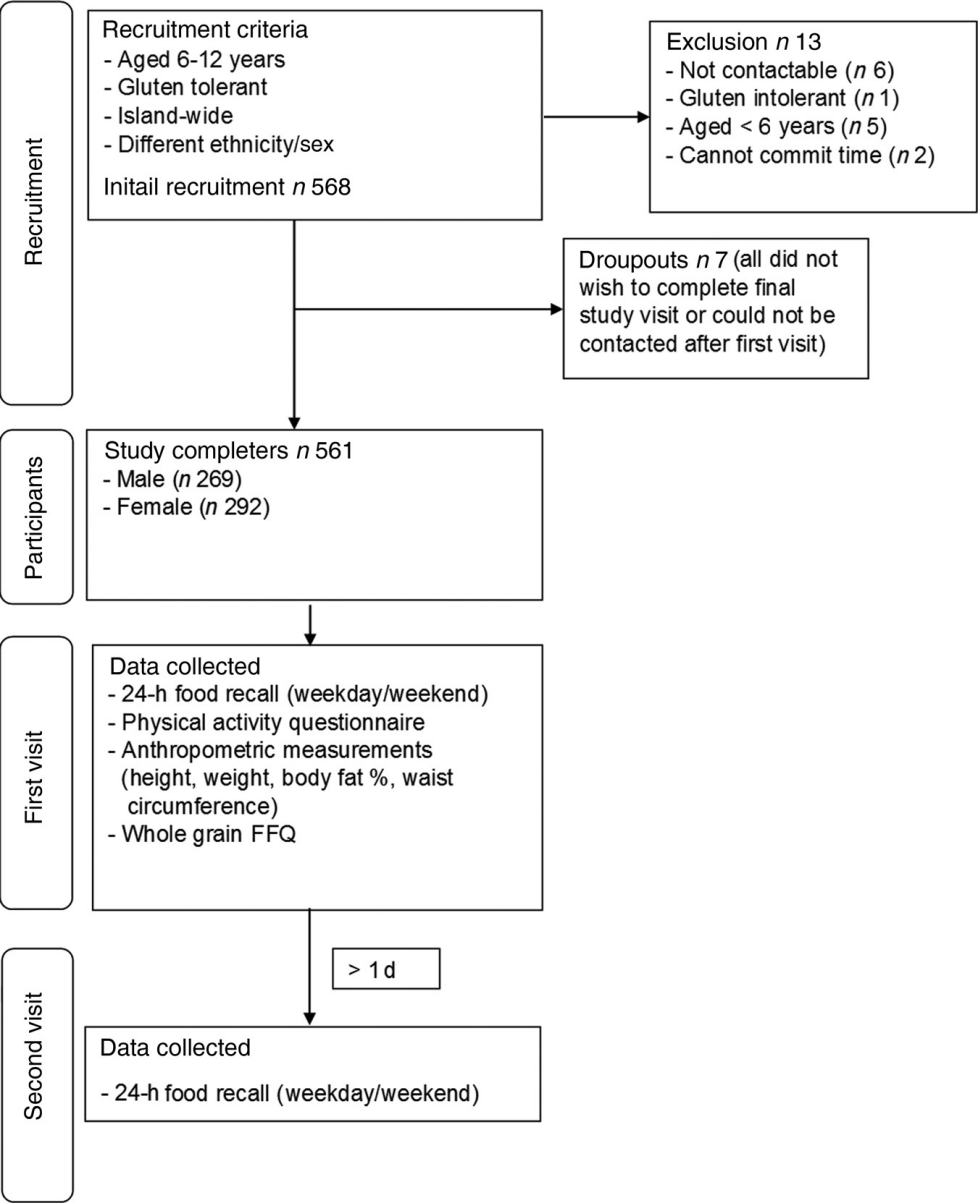
Overview of participants’ recruitment, selection and study design.

Participants were initially sought by postal invitation from a representative sample of addresses provided by the Singapore Department of Statistics. As a result of a poor response rate (only one positive respondent from 300 approaches over 3 months), participants were subsequently sought by approaching parents within shopping centres around Singapore. This was seen as the best available mode of recruitment, as shopping centres are found in all neighbourhoods in Singapore and are visited by the majority of the population for retail, leisure and food and beverage consumption^(^^29^^)^. In order to best ensure that a representative sample of children was recruited, potential participants were approached in all geographic areas of the island and no more than fifty participants were recruited from a single shopping centre. A range of different types of shopping centre were targeted, from those linked to public transport interchanges, to small neighbourhood settings and larger, modern shopping malls to minimise the risk of over- or under-representing demographics. Further participants were also recruited by snowball sampling of friends and colleagues of the initial participants. The demographics of the final study sample were compared with census data on geographical location, sex and ethnicity to evaluate the representativeness of this sampling method. Recruitment outcomes are outlined in Fig. 1.

Inclusion criteria for this study consisted of young children aged between 6 and 12 years old (age on the date of the first visit). Potential participants were excluded if they reported an allergy or intolerance to grain products during the consenting procedure. This study was conducted according to the guidelines laid down in the Declaration of Helsinki and all procedures involving human subjects/patients were approved by the Newcastle University Faculty of Science, Agriculture and Engineering Research Ethics Committee. Full, written informed consent was obtained from both participants and their parents. The most conservative sample size calculation for statistical power would estimate that a sample size of 384 would adequately model the entire population of Singaporean children within this age range (at least 50 000)^(^^30^^)^. Therefore, a sample of 600 participants was initially targeted with the expectation that drop-out rates could be as high as 15 %.

### Dietary intake assessment

Adults accompanied by children were approached in shopping centres by trained researchers. The study was explained verbally and an information sheet given. Following obtaining consent, two home visits were organised with the participant to collect dietary, anthropometric and physical activity information. During the first visit, participants were interviewed to assess their dietary intake by 24-h food recall using a five-step, multiple-pass, method by four trained researchers in the presence of parents or caregivers. Briefly, the researcher would (a) ask the child to recall their dietary intake, (b) discuss any items that might have been forgotten, then (c) go through each meal for additional detail (e.g. amount or brands consumed). This would then be supported by the parent/caregiver or, where necessary by (d) checking brand information or portion sizes of food items consumed in the home. The final step (e) was a review of all the foods and eating occasions to limit the potential for missed items. Physical activity habits were assessed using the International Physical Activity Questionnaire as previously described^(^^31^^,^^32^^)^. Each child participant was interviewed individually before getting assistance from their parent/caregiver. At the same visit, measurements of height and weight (to calculate BMI) and waist circumference were also collected using standardised protocols.

A second visit (on a non-consecutive day) to the household was made to collect an additional 24-h dietary recall so that data were collected for one weekday and one weekend day to give a better representation of the typical consumption pattern of the participants^(^^33^^)^. Dietary recall data included description (including food name, brand, time of day, place) and quantities of all food and drink consumed. For both dietary recall occasions, information reported was captured by the interviewers in a hard copy booklet. Interviewers made use of cups, plates, bowls and household utensils of known size to help the participants in recalling the quantity of food eaten in order to improve estimates of portion sizes consumed^(^^34^^)^. All food and beverage items consumed over each day were entered into the dietary analysis program WinDiets for each participant. After data entry, all foods consumed were sorted into the following commonly consumed major food groups (by four trained researchers): cereals and cereal products; milk and milk products; eggs and egg dishes; fats and spreads; meat and meat products; meat alternatives and vegetable proteins; fish and fish dishes; salad and vegetables; fruit; savoury snacks; sugar preserves and confectionery; nuts and seeds; and dietary supplements. These commonly consumed food groups were further broken down into thirty-five main food subgroups (e.g. bread, rice, pasta, beef, pork, fish and cheese). The categorisation of food items was subsequently checked independently by a single, experienced researcher at the data analysis stage. Weekend and weekday data from dietary recalls were used to estimate weekly intake before calculating a daily average intake for nutrients and food items.

### Identification of whole-grain foods

Each of the commonly consumed food groups was checked for presence of foods containing cereal grains, eliminating milk and milk products, fats and spreads, salad and vegetables, fruit, sugar preserves and confectionery and dietary supplements. Cereal grains identified in the remaining foods were defined as whole grain or non-whole grain using a publication of whole-grain ingredients^(^^35^^)^, with the exception of buckwheat, which has since been identified as a whole-grain ingredient^(^^36^^)^. Whole-grain ingredients identified were brown rice, red rice, buckwheat, whole corn/maize, rolled oats, oatmeal, wholemeal and whole/whole-grain wheat, The whole grain content of foods identified with whole-grain ingredients were cross-checked with a list of food codes and names for which whole grain DM percentage had been previously calculated^(^^37^^)^. Where foods (including regional foods) could not be found on this list they were estimated using nutrient data from the Singapore Government HPB or product-specific information from manufacturers. One food, oatmeal biscuits, had a different composition to that listed in Jones *et al*.^(^^37^^)^ and were more similar to those described in a list of US whole-grain foods which was used instead^(^^38^^)^. No minimum cut-off of level of whole grains was considered for whole-grain foods.

### Estimating whole grain intake

Whole grain intake for each study participant was calculated by identifying each whole-grain food product consumed over the 2 d (one weekday and one weekend day) of dietary recall. The total weight (grams) of each whole-grain product identified was multiplied by the food-specific whole-grain content percentage to give grams of whole grain for each whole-grain food consumed. The grams whole grain consumed were then totalled for all whole-grain foods eaten during each day. To give a representative whole grain intake over a week, intake on the weekday was multiplied by 5 and added to the intake on the weekend day multiplied by 2. Finally, whole grain intake per d was calculated by dividing the weekly intake by 7 to give a representative intake per d of the weekly dietary pattern. Whole grain consumers were defined as any participant who has consumed a whole-grain food at least once over the dietary recall. Whole grain consumers were categorised into tertiles of intake per d and also into groups of servings per d. A serving of whole grain was defined as 16 g/d in line with the United States Department of Agriculture Dietary Guidelines for Americans^(^^15^^)^, where three servings (‘ounce-equivalents’) are equivalent to 48 g of whole grain – a daily amount associated with improved health outcomes^(^^36^^)^. Although lower levels are recommend for most US children, this value (48 g/d) was adopted for this study due to the population study evidence linking this level of habitual whole grain intake with improved health outcomes^(^^2^^)^.

### Statistical analysis

Whole grain intake was summarised, for the total population and consumers only, using medians and IQR due to the skew of the data. Whole grain intake, for the total population and consumers only, was also summarised by sex, age group, ethnicity, region, dwelling type and BMI category and tested using the non-parametric Mann–Whitney rank-sum test (for sex and age group) and Kruskal–Wallis tests. Mean intakes of foods (g/d) and mean BMI, waist circumference and physical activity level values were compared between non-consumers and across tertiles of intake using *t* tests and non-parametric tests for trend. A *P* value of less than 0·05 was considered significant. All statistical analyses were carried out using Stata version 12 (StataCorp LP) and Prism version 6.07 (Graphpad).

## Results

### Participant demographics

Details of the outcomes of participant recruitment, exclusion and drop-out are included in Fig. 1, with demographic details of the participants provided in Table 1. There was a slightly higher percentage of females (52 %) than males. When compared with the geographical spread of population distributed in the five different regions of Singapore (North: 13·4 %; East: 18·4 %; North-East: 19·8 %; Central: 24·6 %; West: 23·7 %) and ethnicity (Chinese: 77·4 %; Malay: 10·3 %; Indian: 10·0 %; other ethnic background: 2·3 %) by *χ*^2^ test for trend, there were no significant differences (*P* > 0·05 for geographical and ethnic distribution compared with previously published estimates of ethnic and geographical distribution^(^^39^^,^^40^^)^). The majority of participants (93 %) resided in either Housing Development Board (public housing) flats (*n* 301) or condominiums (*n* 203).

**Table 1.** Demographic overview of the study participants (Number of subjects and percentages)

	Age (years)							
	6		7–9		10–12		Total	
	*n*	%	*n*	%	*n*	%	*n*	%
Sex								
Male	44	7·8	133	23·7	92	16·4	269	48
Female	37	6·6	164	29·2	91	16·2	292	52
Total	81	14·4	297	52·9	183	32·6	561	100
Ethnicity								
Chinese	56	10·0	238	42·4	140	25·0	434	77
Malay	11	2·0	25	4·5	22	3·9	58	10
Indian	12	2·1	25	4·5	19	3·4	56	10
Others	2	0·4	9	1·6	2	0·4	13	2
Region								
North	14	2·5	53	9·4	37	6·6	104	19
East	17	3·0	54	9·6	27	4·8	98	17
North-East	22	3·9	106	18·9	57	10·2	185	33
Central	13	2·3	38	6·8	19	3·4	70	12
West	15	2·7	46	8·2	43	7·7	104	19
Type of housing								
Public	58	10·3	245	43·7	18	3·2	321	57
Condominium	13	2·3	34	6·1	156	27·8	203	36
Landed property	3	0·5	6	1·1	2	0·4	11	2
Semi-detached	0	0·0	4	0·7	3	0·5	7	1
Terrace	6	1·1	8	1·4	2	0·4	16	3
Conservation flat	1	0·2	0	0·0	2	0·4	3	1

### Whole-grain foods

Details of the whole-grain foods identified in dietary recall are presented in Table 2, as well as information on whole-grain ingredients and content. A total of twenty-two different whole-grain food items were consumed and sorted into seven food groups: pasta/noodles, rice, bread, ready-to-eat breakfast cereals (RTEBC), hot cereal, sweet snacks and savoury snacks. There were a total of 346 occasions of consumption of whole-grain food items over the 2 d of dietary recall data collection. The most commonly consumed whole-grain food group was rice, accounting for 29·5 % of all eating occasions, followed by breads (28·9 % of all eating occasions) and whole-grain RTEBC (18·8 % of all eating occasions) (see Table 2).

**Table 2.** Whole-grain (WG) foods items noted to be consumed by participants using 24 h dietary recalls

						WG eating occasions per food item		WG eating occasions per food group	
Food group	WG food products	Grain source	Estimated portion weight (g)*	WG/ 100 g	g WG/ portion	%	*n*	%	*n*
Pasta and noodles								6·9	24
	Brown rice beehoon/vermicelli	Rice	176·0	26·1	45·9	1·4	5		
	Soba noodles, cooked	Buckwheat	137·5	30·9	42·5	4·0	14		
	Wholemeal pasta, cooked†	Wheat	222·0	30·9	68·6	1·4	5		
Rice								29·5	102
	Brown or red rice, cooked‡	Rice	195·0	34·0	66·3	27·5	95		
	Brown rice porridge	Rice	256·0	6·5	16·6	2·0	7		
Bread								28·9	100
	Mixed grain bread, WG	Wheat	36·0	49·0	17·6	3·2	11		
	Oat and honey bread	Oats	36·0	11·8	4·2	2·0	7		
	Wholemeal bread	Wheat	36·0	54·8	19·7	23·7	82		
RTEBC								18·8	65
	Brown rice cereal	Rice	30·0	22·0	6·6	0·9	3		
	Granola	Mixed	40·0	48·8	19·5	0·9	3		
	RTEBC1	Wheat	30·0	24·0	7·2	0·9	3		
	RTEBC2	Mixed	30·0	25·9	7·8	2·6	9		
	RTEBC3	Wheat	30·0	25·0	7·5	9·5	33		
	RTEBC4	Wheat	30·0	24·3	7·3	3·5	12		
	RTEBC5	Mixed	48·8	40·0	19·5	0·6	2		
Hot cereal								6·1	21
	Oatmeal porridge	Oats	121·5	11·0	13·4	6·1	21		
Sweet snacks								8·4	29
	Digestive biscuits	Wheat	10·0	13·8	1·4	1·2	4		
	Muesli bar	Mixed	28·0	40·1	11·2	0·9	3		
	Oatmeal biscuits	Oats	14·0	14·3	2·0	2·3	8		
	Popcorn, sweet or salted	Maize	43·5	61·4	26·7	4·0	14		
Savoury snacks								1·4	5
	Tortilla chips	Maize	50·0	63·4	31·7	1·2	4		
	Whole-wheat crackers	Wheat	10·0	20·8	2·1	0·3	1		

RTEBC, ready to eat breakfast cereal.

* Estimated portion weight data are representative of portion sizes consumed in the study.

† Wholemeal pasta originally calculated from dry weight and subsequently converted to cooked weight due to food composition code used in dietary analysis.

‡ One participant presented rice and dry weight portion size and is included in the cooked rice list.

### Whole grain intake

There were 215 (38·3 %) children who ate whole grain at least once over the 2 d of dietary recall and were classified as ‘whole grain consumers’. Of these, 150 (26·7 %) consumed whole grains on weekdays, 114 (20·3 %) at the weekend and forty-nine on both days. The patterns of whole grain intake across different demographics are presented in Table 3. Median intake of whole grains was estimated to be 0·0 (interquartile range (IQR) 0·0–9·4) g/d. The median intake of whole grains in consumers only was 15·3 (IQR 5·4–34·8) g/d. Compared with other ethnicities (where around 40 % of individuals were whole grain consumers), a significantly lower proportion of ethnic Malay children were whole grain consumers (seven out of fifty-eight) compared with non-Malay participants (208 out of 503; *P* < 0·0001 by χ^2^ test).

**Table 3.** Whole grain consumption across the participant demographic (Number of subjects and percentages, medians and interquartile ranges (IQR))

	All participants				Whole grain consumers				
	*n*	%	Median	IQR	*n*	% of consumers	Median	IQR	*P*
Total population	561	100	0·00	0·00–9·39	215	38·3	15·31	5·36–34·78	
Sex									
Male	262	46·7	0·00	0·00–8·40	103	39·3	13·99	5·19–35·22	0·61*
Female	299	53·5	0·00	0·00–9·89	112	37·5	16·51	5·96–33·18	
Age group									
6–9 years	378	67·4	0·00	0·00–10·7	154	40·7	14·04	6·36–33·33	0·75*
10–12 years	183	32·6	0·00	0·00–4·46	61	33·3	16·40	4·46–34·78	
Region									
North	105	18·7	0·00	0·00–6·72	34	32·4	17·63	8·04–28·34	0·46†
East	99	17·6	0·00	0·00–9·39	42	42·2	10·53	4·86–33·33	
North-East	186	33·2	0·00	0·00–4·55	58	31·2	11·82	5·20–34·78	
Central	66	11·8	0·00	0·00–14·08	32	48·5	15·19	8·12–32·82	
West	105	18·7	0·00	0·00–15·31	49	46·7	16·44	7·86–55·21	
Ethnicity									
Chinese	434	77·4	0·00	0·00–11·2	179	41·2	16·51	5·36–36·68	0·23†
Malay	58	10·3	0·00	0·00–0·00	7	12·0	11·08	3·42–17·11	
Indian	56	10·0	0·00	0·00–7·59	24	42·9	9·39	5·90–20·82	
Others	13	2·3	0·00	0·00–17·07	5	38·5	25·63	17·07–87·87	
Housing type									
HDB	459	81·8	0·00	0·00–7·15	162	75·4	15·70	5·20–33·03	0·68†
Condominium	65	11·6	0·00	0·00–12·73	32	14·9	14·02	8·45–49·34	
Other‡	37	6·6	0·96	0·00–17·11	21	9·8	16·51	1·92–39·79	
BMI category§									
Underweight	54	9·6	0·00	0·00–3·90	18	33·3	8·92	3·90–23·49	0·29†
Normal weight	428	76·3	0·00	0·00–10·53	169	39·5	15·31	5·55–32·61	
Overweight	55	9·8	0·00	0·00–2·38	16	29·1	22·61	8·00–70·70	
Obese	24	4·3	0·72	0·00–29·36	12	50·0	29·36	7·27–62·72	

HDB, Housing and Development Board (public housing).

* Test of significant differences in intakes (for consumers only) between groups using the Mann–Whitney test.

† Test of significant differences in intakes (for consumers only) between groups using the Kruskal–Wallis test (unadjusted for confounding factors).

‡ Other includes houses and other landed properties.

§ BMI category defined by age- and sex-specific percentiles.

The greatest contribution of foods to whole grain intake per d came from rice (40·1 %) followed by breads (23·7 %), pasta/noodles (19·7 %), RTEBC (8·2 %), sweet snacks (3·3 %), savoury snacks (2·9 %) and hot cereals (oatmeal, 2·1 %). Across all foods identified, the main whole grain source contributing to whole grain intake was rice accounting for 42·0 %, coming from cooked brown/red rice, brown rice beehoon and brown rice cereal. Wheat accounted for 39·4 % of all whole-grain foods consumed, coming from wholemeal/whole-grain breads, wholemeal pasta, RTEBC, biscuits and crackers. Buckwheat consumption (8·7 % of all whole-grain foods) came from soba noodles. Oat consumption (5·3 % of all whole-grain foods) came from oat bread, oatmeal porridge, oatmeal biscuits and RTEBC. Finally, maize/corn consumption (4·7 % of all whole-grain foods) came from popcorn and tortilla chips. Just over 80 % of eating occasions of whole-grain food items took place at home (either the home of the participants or their friends and relatives). Consumption of whole-grain foods was most common out of home at school (45 % of out-of-home consumption occasions; data not shown).

The majority of whole grain intake per d was consumed at breakfast (38·2 %) and dinner (35·6 %), as outlined in Table 4 and on weekdays (63 %) compared with weekends (37 %). During breakfast the majority of whole grain was consumed as breads (50·3 %), at lunchtime as rice (51·2 %), at tea breaks as pasta/noodles (37·3 %) and dinner as rice (79 %). Finally, during supper the majority of whole grain was consumed as savoury snacks (42·9 %). The majority of whole grain intake took place in the home (87·5 %) with an additional 2·8 % being consumed in a friend's or relative's home. A further 8·4 % of whole grain intake happened at school with the remaining 1·3 % of whole grain intake occurring infrequently at cafés, restaurants, food courts, shopping centres, community buildings, on the move and at cinemas.

**Table 4.** Percentage contribution of whole grain (WG) intake per d by meal occasion and food group

		Food group (% of WG intake per d)						
Meal occasion	% of WG intake per d	Pasta/noodles	Rice	Bread	RTEBC	Hot cereals	Sweet snacks	Savoury snacks
Breakfast (06.00–11.00 hours)	38·2	19·3	6·5	50·3	14·1	4·7	1·0	4·0
Lunch (11.00–14.00 hours)	16·7	23·6	51·2	6·6	3·2	1·2	12·3	1·8
Tea break (14.00–18.00 hours)	6·8	37·3	4·9	22·2	14·3	1·0	20·3	0·0
Dinner (18.00–21.00 hours)	35·6	15·6	79·0	2·8	1·7	0·0	0·8	0·0
Supper (21.00–00.00 hours)	2·8	0·0	0·0	27·0	16·1	8·6	5·4	42·9

RTEBC, ready to eat breakfast cereals.

**Fig. 2.**
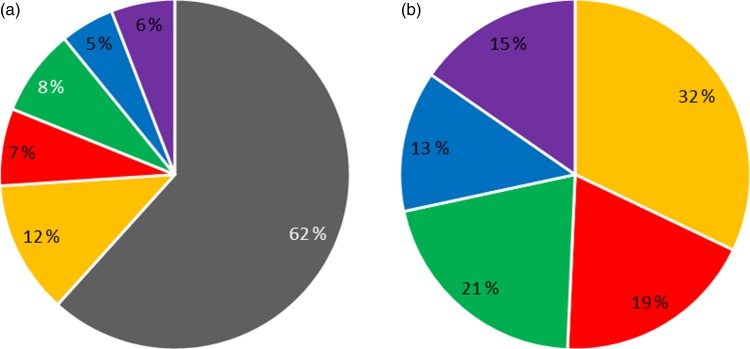
Consumption of whole grains in (a) all participants and (b) whole grain consumers only. 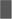 = 0, 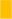 = < 8, 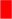 = 8–16, 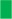 = 16–32, 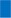 = 32–48 and 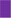 = > 48 g/d.

A large proportion of the whole grain consumers had relatively low absolute intakes of whole grains in comparison with the US standard 16 g serving of whole grain. Of whole grain consumers, 15 % consumed 48 g/d or more of whole grains (Fig. 2) which equates to approximately 5·9 % of the total participants who were surveyed.

### Dietary intake, fatness and lifestyle in whole grain consumers

Dietary intakes of milk and milk products, cheese, fruit, vegetables, whole-grain bread and RTEBC were significantly higher for whole grain consumers compared with non-consumers and across increasing tertiles of whole grain intake (Table 5). No significant differences were seen between consumers and non-consumers or across increasing whole grain intake for BMI, waist circumference and physical activity level.

**Table 5.** Intakes of other food groups for non-consumers *v*. consumers and across tertiles of whole-grain (WG) intake from 24-h recall (Mean values)

	Non-consumers (*n* 346)	All WG consumers (*n* 215)	P*	Tertile 1 (*n* 71)	Tertile 2 (*n* 72)	Tertile 3 (*n* 72)	*P*†
WG intake (g/d)	0	25·98	<0·001	3·82	15·42	58·39	
BMI (kg/m^2^)	17·36	17·37	0·999	16·95	17·02	18·12	0·523
Waist circumference (cm)	60·68	60·91	0·776	60·62	60·45	61·67	0·601
Physical activity level	1·91	1·93	0·136	1·93	1·94	1·93	0·281
Food group intakes (g/d)							
Milk and milk products	76·69	125·54	<0·001	148·07	116·92	111·93	<0·001
Cheese	2·81	4·63	0·033	3·23	2·93	7·72	<0·001
Yogurts and dairy puddings	2·71	7·11	0·064	10·16	3·51	7·69	0·001
Eggs and egg dishes	25·66	25·68	0·992	19·15	31·37	26·44	0·894
Fats and spreads	0·95	1·24	0·286	0·84	1·13	1·76	0·068
White meat (chicken, duck, frog)	59·71	59·32	0·942	72·77	49·18	56·21	0·477
Red meat (beef, lamb, pork)	37·51	44·00	0·123	38·55	45·90	47·46	0·063
Fish and fish dishes (including shellfish)	59·21	53·79	0·316	46·91	62·64	51·74	0·681
Meat alternatives/vegetable proteins	12·97	11·75	0·671	10·10	7·21	17·92	0·918
Fruit	56·47	88·96	<0·001	63·32	108·76	94·45	<0·001
Vegetables (including salad)	52·91	85·59	<0·001	59·53	91·69	105·19	<0·001
Potatoes	18·34	18·17	0·952	17·69	16·15	20·67	0·795
Pasta, rice and other cereals	354·03	342·17	0·414	300·73	374·06	351·13	0·477
White bread	37·32	31·35	0·134	36·47	28·75	28·91	0·031
WG bread	0·00	10·23	<0·001	1·52	11·61	17·45	<0·001
Ready-to-eat breakfast cereals	1·60	8·98	<0·001	10·40	7·04	9·52	<0·001
Snacks (biscuits, crackers, crisps, snacks)	16·23	13·54	0·300	13·38	9·96	17·26	0·179
Buns, cakes, pastries, pancakes	45·02	44·44	0·918	50·00	49·51	33·88	0·590
Desserts	4·02	8·27	0·082	6·97	10·87	6·96	0·024
Confectionery	4·65	4·55	0·931	4·33	3·47	5·86	0·301

* *t* Test of mean difference between non-consumers *v*. consumers.

† Test for trend across non-consumers and tertiles of intake. *P* < 0·05 shows significant difference/trends. No comparisons have been adjusted for confounding factors.

## Discussion

To the authors’ knowledge, this is the first study to estimate intake of whole grains by Singaporean children. Whole-grain food consumption was infrequent in the study population, with only 38 % of participants classified as whole grain consumers. The majority of whole grain consumers ate low overall amounts of whole grains, with just 19 % of all participants consuming more than the equivalent amount of whole grains found in a slice of bread (16 g/d; Fig. 2).

In Singapore, the National Nutrition Survey 2010 suggested that frequency of whole-grain consumption was approximately 0·76 servings of whole grains per d in adults, which is approximately 24 g/d of whole grains consumed. A more recent study in pregnant women in Singapore suggested a similarly low intake of whole grains as seen in our study population, with median intake of whole grains estimated to be 0 (IQR 0–9·0) g/d from 24-h dietary recall methods^(^^41^^)^. Recent studies carried out in France, Italy, the UK and Ireland and have also reported low whole grain consumption in children and adolescents^(^^22^^,^^42^^,^^43^^)^. Median whole grain intakes in our study were similar to levels reported in the UK and Ireland; however, in these countries almost 90 % of children were consumers of whole grain compared with only 38 % in our study. We are aware of only one other study in the region which assessed whole grain intake in children, which was conducted in Malaysia^(^^43^^)^. Whole grain consumption in Malaysian children was much lower than in Singapore (median intake equivalent to 7 g/d in consumers), with only about a quarter of children having reported consumption of whole grain. These findings could be related to differences in dietary practices between the two countries or could highlight the success of health promotion strategies aimed at increasing whole grain availability and intake in Singapore^(^^17^^,^^19^^,^^44^^)^.

The whole-grain foods most frequently consumed by our participants were brown rice, wholemeal breads and RTEBC. In several European countries and the USA, bread and RTEBC are among the top two contributors to whole grain intakes in children; however, brown rice only made a minimal contribution. Notably, in Malaysia which has similar dietary patterns to Singapore, rice and bread contributed <2 % to total whole grain intakes. Breads and other whole-grain foods items may be frequently consumed within the study place as Singaporean schools provide whole grain items (e.g. brown rice mixed with white rice, wholemeal bread) in canteens as part of a Ministry of Education initiative within primary schools^(^^44^^)^. Lack of access or availability to whole-grain foods has previously been cited to be a major limiting factor to whole-grain food consumption^(^^45^^,^^46^^)^. The study place-based initiatives appear to have been somewhat successful as the largest proportion of out-of-home whole-grain food consumption events were within the school setting. However, this only accounted for a low overall percentage (8·4 %) of the total number of whole-grain eating events. The vast majority of whole grains consumed (>80 %) were within the home. A preliminary qualitative study in young Singaporean adults living at home has highlighted that, alongside limited access to whole-grain foods in and out of the home, further barriers to consumption, including poor taste expectations and limited food-purchasing decisions, should also be considered in order to increase whole grain intake within children as individuals or at a population level. Consumption in the home is also likely to be as part of a communal meal where provision of foods that are acceptable to all family members could become a limiting factor to consumption of whole grains^(^^47^^,^^48^^)^.

A lower percentage of Malay ethnic children appeared to consume whole grains than other ethnicities within the present study. A recent study on whole grain intake in Malaysian children noted that the overall percentage of whole grain consumers (25 %) was slightly lower than in the present study (38 %), but there did not appear to be a major difference in the percentage of ethnic Malay, Chinese or Indian children who reported eating whole grains^(^^43^^)^. These findings highlight a need for further, qualitative research to consider how best to target increased whole-grain food intake in Singaporean Malay children.

Increasing intake of whole grains at a population level is a challenging proposition as it involves the participation of multiple stakeholders. Such initiatives appear to have been successful in Denmark, where whole grain consumption at a population level has increased by approximately 75 % in the first 6 years of the Whole Grain Partnership^(^^49^^)^. Further efforts should be made to substitute all whole-grain foods into the normal dietary practices of children in the home, at study and when consuming food out-of-home. Inputs from multiple stakeholders, including parents, children, public health agencies, academic institutions, whole-grain food manufacturers and food vendors of all types (from specialist food stalls to large supermarkets) in Singapore will be necessary to improve the chances of successfully increasing whole grain intake in young Singaporeans. Cross-sectional studies in children from Europe and the USA have demonstrated that consumption of whole grain at a daily amount similar to or less than that noted in consumers in the present study was associated with significantly higher daily intakes of dietary fibre and several B vitamins and some minerals, such as Mg, Fe, P and K^(^^22^^,^^23^^,^^50^^,^^51^^)^. Therefore, encouraging current non-consumers to include just one serving of whole-grain food per d such as a bowl of wholegrain RTEBC, a portion of brown rice or a slice of wholemeal bread could have a positive impact on nutritional intake. It has also been observed by others that consumers of whole grain tend to consume higher amounts of other micronutrient-dense food groups such as fruit and vegetables and dairy products^(^^22^^,^^23^^,^^49^^,^^50^^)^, a finding which was corroborated in the present study.

The authors believe that the cross-sectional dataset of dietary habits in Singaporean children is novel and will provide a valuable asset for further analyses. Demographic data suggest that our final sample was representative of the population of Singaporean children (Table 1). However, our original plans for recruitment through postal invitation would have had less potential for selection bias for potential confounding factors not assessed within this study (e.g. family education level, socio-economic status). Based on the limited number of applicants who were recruited through this method, it would not have been possible to recruit as large a cross-section of participants even if all children in Singapore aged 6 to 12 years were approached. The authors therefore feel that the approach taken was valid and that the possible reason that the number of postal respondents was so low was due to a lack of familiarity with the research institution involved. The authors also note that while the dataset was relatively small (561 completers), the drop-out rate was very low (seven out of 568) which further highlights an acceptable choice of methodological approach and study execution. Due to the nature of the recruitment process, no data were collected from non-responders and those that declined to participate when approached in shopping malls. Although we are confident that the distribution of participants was representative of the whole Singaporean population, without this information this cannot be confirmed and is a potential limitation of the study.

Previous studies have highlighted that the multipass dietary recall is a valid method of estimating habitual dietary intake^(^^52^^)^. Within the present study, we repeated data collection on a weekday and a weekend day, which further improves the validity of the findings^(^^53^^)^. While increasing the number of repeats of the 24-h dietary recall method is likely to improve the reliability of overall data, it also increases participant burden and has the potential to limit study completion. Although weighed, 7-d food diaries are considered the ‘gold standard’ in estimation of dietary intake^(^^54^^)^, they are associated with high participant burden and are not widely used in studies in children. The dietary recall method used in this study therefore probably represents the most appropriate method of estimation of dietary intake available, despite the potential for issues with recall accuracy in younger children^(^^55^^)^ and the potential for this method to collect a snapshot of dietary intake that is not representative of habitual consumption patterns.

The current project required collation of a wide range of food portion sizes and nutrient composition data that are not currently available in commonly used food databases. Food in Singapore is available from a wide number of food manufacturers worldwide and includes a number of ethnic Chinese, Malay and Indian foods as well as other items that are specific to Southeast Asia. These data were sourced from existing nutritional databases for Singapore^(^^56^^)^, Malaysia^(^^57^^)^ and Hong Kong^(^^57^^)^, with other items added based on food manufacturers’ product information or recipe information. As comparable data do not exist in other international food composition tables, the authors feel that this represents the best available approach but acknowledge that this is a limitation of the study.

Current recommendations for children in Singapore are to include two or three servings of whole grains per d^(^^18^^)^. However, the suggested servings of whole-grain foods are not equivalent in terms of grams of whole grains provided^(^^58^^)^. For example, two slices of wholemeal bread would equate to 33 g of whole grain, assuming a slice is 30 g, whereas, four whole-wheat biscuits would equate to 8 g of whole grain, assuming a biscuit is 10 g. Due to these variations in whole grain quantities we chose one serving of whole grains to be 16 g (as described above) since individuals that consume at least 48 g (three servings) regularly are believed to be at the lowest risk for CVD^(^^59^^)^. This highlights a global challenge with the standardisation of assessment of whole grain intake^(^^13^^)^, which has implications for researchers and those developing health promotion messages. A key element in improving the current situation would be to develop international databases of whole grain content in foods.

### Conclusions

Few Singaporean children appear to habitually consume whole grains and intake in the majority of consumers is low. Continued efforts are needed to raise knowledge of the importance of whole grains in children's diets and to improve the awareness of available sources of wholegrain foods in Singapore. Longer-term research is required to better understand the barriers to whole grain consumption in this age group so that strategies can be designed to encourage increased whole grain consumption among all children.
